# An Evaluation of the High Dose Antipsychotic Therapy Patients on the General Adult Inpatient Wards and the Psychiatric Intensive Care Unit in Mersey Care NHS Foundation Trust – Is Clozapine Being Considered in Each Case?

**DOI:** 10.1192/bjo.2024.581

**Published:** 2024-08-01

**Authors:** Declan Hyland, Roopa Singh, Kerry Dainton

**Affiliations:** Mersey Care NHS Foundation Trust, Liverpool, United Kingdom

## Abstract

**Aims:**

High Dose Antipsychotic Therapy (HDAT) should only be used in exceptional circumstances, as there is little evidence to suggest that higher than recommended doses of antipsychotics are more clinically effective than standard doses, with potential side effects being greater. In practice, there are several clinical scenarios where HDAT may be prescribed and the potential benefits must outweigh the potential risks. NICE guidelines for psychosis and schizophrenia advise that dosages outside the range given in the British National Formulary should be justified and recorded.

This audit aimed to determine whether the option of clozapine is being considered in those patients on the 16 general adult inpatient wards and Psychiatric Intensive Care Unit (PICU) in Mersey Care NHS Foundation Trust who are prescribed HDAT.

**Methods:**

A list of all inpatients admitted to the 16 general adult inpatient wards and to the PICU in the Trust between 17^th^ and 20^th^ of July 2023 was obtained. The electronic prescription record for each patient was scrutinised to determine whether the patient was subject to HDAT and, if so, whether there was documentation in the patient's electronic record that the option of treatment with clozapine was considered. The authors also wished to determine whether, in those HDAT patients in whom clozapine was considered, the rationale for it not being pursued as a treatment option was documented.

**Results:**

29 inpatients on the 16 general adult wards and on the PICU were prescribed HDAT. In 9 (39%) of the HDAT patients, the option of treatment with clozapine was considered. It was documented for 6 of the HDAT patients that the option of clozapine was not applicable. Of the 9 HDAT patients that had a trial of clozapine considered, all of them had documented evidence of the decision in their electronic record. Four of the 9 patients accepted the trial of clozapine, 5 did not accept/it was deemed not appropriate. Of the 5 patients who did not accept the trial of clozapine or were deemed not appropriate, the rationale was documented for each patient.

**Conclusion:**

Given the lack of recommendation and evidence base to prescribe HDAT, the option of clozapine, if appropriate, should always be considered in any patients in whom HDAT is being considered or required. There may be barriers to clozapine being chosen – both patient-related and clinician-related. Any such barriers should be explored and addressed to ensure that treatment-resistant patients are commenced on clozapine without unnecessary delay.

